# Effects of confounding and effect-modifying lifestyle, environmental and medical factors on risk of radiation-associated cardiovascular disease

**DOI:** 10.1186/s12889-024-18701-9

**Published:** 2024-06-15

**Authors:** Mark P. Little, Marjan Boerma, Marie-Odile Bernier, Tamara V. Azizova, Lydia B. Zablotska, Andrew J. Einstein, Nobuyuki Hamada

**Affiliations:** 1 Radiation Epidemiology Branch, National Cancer Institute, Room 7E546, 9609 Medical Center Drive MSC 9778, Bethesda, MD 20892-9778 USA; 2 Faculty of Health and Life Sciences, Oxford Brookes University, Headington Campus, Oxford, OX3 0BP UK; 3 Division of Radiation Health, University of Arkansas for Medical Sciences, Little Rock, AR USA; 4 Institut de Radioprotection et de Sureté Nucléaire, Fontenay Aux Roses, France; 5 Clinical Department, Southern Urals Biophysics Institute, Chelyabinsk Region, Ozyorskoe Shosse 19, Ozyorsk, 456780 Russia; 6 Department of Epidemiology and Biostatistics, School of Medicine, University of California San Francisco, 550 16th St 2nd floor, San Francisco, CA 94143 USA; 7 Seymour, Paul, and Gloria Milstein Division of Cardiology, Department of Medicine, and Department of Radiology, Columbia University Irving Medical Center/New York-Presbyterian Hospital, New York, NY USA; 8 Biology and Environmental Chemistry Division, Sustainable System Research Laboratory, Central Research Institute of Electric Power Industry (CRIEPI), 1646 Abiko, Chiba 270-1194, Japan

**Keywords:** Cardiovascular disease, Ionising radiation, Heart disease, Stroke, Coronary heart disease, Confounding

## Abstract

**Background:**

Cardiovascular disease (CVD) is the leading cause of death worldwide. It has been known for some considerable time that radiation is associated with excess risk of CVD. A recent systematic review of radiation and CVD highlighted substantial inter-study heterogeneity in effect, possibly a result of confounding or modifications of radiation effect by non-radiation factors, in particular by the major lifestyle/environmental/medical risk factors and latent period.

**Methods:**

We assessed effects of confounding by lifestyle/environmental/medical risk factors on radiation-associated CVD and investigated evidence for modifying effects of these variables on CVD radiation dose–response, using data assembled for a recent systematic review.

**Results:**

There are 43 epidemiologic studies which are informative on effects of adjustment for confounding or risk modifying factors on radiation-associated CVD. Of these 22 were studies of groups exposed to substantial doses of medical radiation for therapy or diagnosis. The remaining 21 studies were of groups exposed at much lower levels of dose and/or dose rate. Only four studies suggest substantial effects of adjustment for lifestyle/environmental/medical risk factors on radiation risk of CVD; however, there were also substantial uncertainties in the estimates in all of these studies. There are fewer suggestions of effects that modify the radiation dose response; only two studies, both at lower levels of dose, report the most serious level of modifying effect.

**Conclusions:**

There are still large uncertainties about confounding factors or lifestyle/environmental/medical variables that may influence radiation-associated CVD, although indications are that there are not many studies in which there are substantial confounding effects of these risk factors.

## Introduction

Cardiovascular disease (CVD) is the leading cause of death worldwide [1–3]. The main independent risk factors for CVD are cigarette smoking, hypertension, diabetes, obesity and elevated total cholesterol or elevated low density lipoprotein (LDL) cholesterol [4–6]. It has been known for some considerable time that high dose radiotherapy is also associated with excess risk of CVD [7, 8]. More recently, it has become clear that there are also radiation-associated excess risks in the Life Span Study (LSS) of the Japanese atomic-bomb survivors [9, 10], and in a number of groups exposed at still lower levels of radiation dose and at lower dose rates [11]. A recent systematic review and meta-analysis of epidemiological studies highlighted evidence of association between radiation exposure and CVD at high dose, and to a lesser extent at low dose, with some indications of differences in risk between acute and chronic exposures [12]. There was inter-study heterogeneity, possibly a result of confounding or modifications of radiation effect by other factors, which complicates a causal interpretation of these findings [12]. Although a number of studies assessed in the previous review adjusted for many of the major lifestyle risk factors, relatively few studies undertook investigation of the modifying effect of these risk factors on the radiation associated CVD.

In this paper we assess effects of confounding by lifestyle, environmental, and medical risk factors, and also investigate evidence for modifying effects of these variables on radiation dose response.

## Methods

The data used are those assembled in a recent systematic review [12]. In brief, the review was conducted, and reported according to PRISMA and registered in PROSPERO (https://www.crd.york.ac.uk/prospero/) (reg. no. 202036). PubMed/MEDLINE, Embase, Scopus, and Web of Science: Core Collection were used to systematically search the literature, with no limits applied (date, language), on 6th October 2022. We excluded animal studies, and any study without an abstract. The database search yielded a total of 15,098 articles.

### Outcomes

CVD was defined as those causes of mortality and incidence with International Classification of Diseases 10th revision (ICD10) codes I00-I99 (or equivalently the ICD 8th revision (ICD8) codes 390–458 or ICD 9th revision (ICD9) codes 390–459).

### Main exposure

Only those studies with individual organ dosimetry that enable estimation of excess relative risk per unit absorbed dose in Gy (ERR/Gy) in relation to heart or brain dose or other closely related tissue doses were used.

### Potentially confounding and effect modifying variables considered

We only used those studies in which there was adjustment for any factor other than the standard demographic risk factors (age, sex, year of birth etc.), and in which ERR/Gy were reported both with and without adjustment, or alternatively in which ERR/Gy were reported of the modifying effect on radiation response of these variables. In other words, to assess potential confounding a relative risk model had been fitted of the form $RR=\exp\lbrack\beta V\rbrack(1+\alpha D)\;$ with adjustment for a potentially modifying variable $V$ and also without adjustment for that potentially modifying variable $V$. A modifying variable in a particular study was any variable $V$ for which had been assessed the interactions with radiation dose, in other words in which a model had been fitted of the form $RR\;=\;1+\alpha D\;\exp\lbrack\beta V\rbrack$ . In some cases the only reported effect was a *p*-value (e.g. of significance of modification). All these studies are listed in Tables 1 and 2.

**Table 1:** Unadjusted or adjusted estimated excess relative risk of cardiovascular diseases in various therapeutically and diagnostically treated groups, exposed at moderate or high radiation doses and high dose rates. All analyses adjust for age

Study	Reference	Organ used	Variables (other than age, sex, year) available to assess possible confounding	Endpoint	Mortality, incidence or prevalence	Unadjusted (or less adjusted) excess relative risk Gy^−1^ (95% CI)	Fully adjusted excess relative risk Gy^−1^ (95% CI)	Notes
Therapeutically treated groups								
Childhood Cancer Survivor Study	Mulrooney et al. [13]	Heart	Smoking, BMI, diabetes, hypertension, dyslipidemia, racial/ethnic group, education, chemotherapy	Heart failure CTCAE v4.03 ≥ 3	Incidence	NA	0.022 (-0.093 to 0.138)^a^	Presented RR of various cardiac endpoints are adjusted for race, BMI, smoking, exercise intensity. Unadjusted analyses not presented. Additional analysis in which cardiotoxic exposures are adjusted for did not appreciably change RR for heart failure, coronary artery disease, valvular disease, pericardial disease, arrhythmia in relation to initial cancer endpoint: change in RR always < 10%
				Coronary artery disease CTCAE v4.03 ≥ 3		NA	0.066 (-0.020 to 0.152)^a^	
				Valvular disease CTCAE v4.03 ≥ 3		NA	0.064 (-0.178 to 0.306)^a^	
				Pericardial disease CTCAE v4.03 ≥ 3		NA	-0.005 (-0.082 to 0.072)^a^	
				Arrhythmia CTCAE v4.03 ≥ 3		NA	0.005 (-0.049 to 0.058)^a^	
French–UK childhood cancer study	Tukenova et al. [14]	Heart	Epipodophyllotoxins, anthracyclines, alkylating agents, vinca alkaloids, antimetabolites, antibiotics	Cardiac disease	Mortality	NA	0.6 (0.2 to 2.5)	Presented ERR is adjusted for alkylating agents, vinca alkaloids, anthracyclines (including dose), antimetabolites. No unadjusted ERR are presented. Radiation dose did not significantly interact with exposure to anthracyclines (*p* > 0.3) or any other type of drug
French (Institut Gustave Roussy) childhood cancer cardiac study	Haddy et al. [15]	Heart	Smoking, BMI, anthracyclines, alkylating agents, vinca alkaloids, epipodophyllotoxins, antimetabolites	Cardiac disease (ICD9 391, 393–397, 410–413, 420, 423–424, 426–428; ICD10 I05–I09, I20–I25, I30–I32, I44–I50): without anthracyclines	Incidence	0.49 (0.26 to 1.3)	NA	ERR are not adjusted for any variable. RR in highest dose group (> 30 Gy) are “similar when the analysis is controlled for smoking status and BMI”
				Cardiac disease: with anthracyclines		0.07 (0.03 to 0.13)	NA	
French Childhood Cancer Study case–control study	Mansouri et al. [16]	Heart	Smoking, BMI, physical activity, anthracyclines, alkylating agents, vinca alkaloids	Heart failure (CTCAE v4.03 grade ≥ 1) with concomitant anthracyclines	Incidence	NA	0.09 (0.02 to 0.22)	ERR were adjusted for splenectomy, type of first malignancy, vinca alkaloids, alkylating agents and other chemotherapy. A modifying effect of anthracycline suggested (but no test of significance). Apart from effect of anthracyclines “no other factors significantly modified the effects of radiation. However, HF risk increased with each category of age, with ERR/Gy values for the MHD of 0.06, 0.33, 0.38 and 0.48 in those aged < 15, 15–25, 25–35, and ≥ 35 years, respectively.”
				Heart failure (CTCAE v4.03 grade ≥ 1) without concomitant anthracyclines		NA	0.44 (0.18 to 1.12)	
Netherlands Hodgkin lymphoma coronary heart disease case–control study	van Nimwegen et al. [17]	Heart EQD2	Smoking, BMI, diabetes, hypertension, hyper-cholesterolemia, physical activity, alkylating agents, procarbazine, vincristine, anthracyclines, splenectomy	Coronary heart disease (angina pectoris, myocardial infarction requiring intervention) CTCAE v4.0 grades ≥ 2	Incidence	NA	0.074 (0.033 to 0.148)	ERR is adjusted for chemotherapy. There is no unadjusted analysis. There is “no evidence for statistically significant modification of the effect of MHD on CHD risk by chemotherapy, sex, cardiovascular disease risk factors, and recent smoking at HL diagnosis.”
Netherlands Hodgkin lymphoma heart failure case–control study	van Nimwegen et al. [18]	Heart EQD2	Smoking, BMI, diabetes, hypertension, hyper-cholesterolemia, physical activity, anthracyclines, splenectomy	Heart failure CTCAE v3.0, v4.0 grades ≥ 2	Incidence	0.038 (-0.001 to 0.146)^b^	NA	Although many adjusting variables are available, only RR for unadjusted analysis are presented. RR in high dose group (≥ 26 Gy) “did not differ significantly according to use of anthracycline chemotherapy (*p* = 0.45 for MHD, 0.09 for MLVD) or with splenectomy (*p* = 0.71 for MHD, 0.62 for MLVD).” No additional significant interactions by sex (*p* = 0.72), age at HL diagnosis (*p* = 0.43), or time since HL diagnosis (*p* = 0.92)
Nordic breast cancer case–control study	Darby et al. [19]	Heart	Smoking, BMI, diabetes, hypertension, analgesic medication, thyroid medication, surgery, HRT, chemotherapy, ovarian ablation, history of IHD or COPD	IHD (ICD10 I20-I25)	Incidence	NA	0.074 (0.029 to 0.145)	ERR was estimated adjusting for cardiac risk factor (history of IHD or other circulatory disease, smoking, BMI, history of COPD, diabetes, analgesic medication). The risk was very “similar for women with and those without cardiac risk factors” (ERR/Gy = 0.074 (95% CI 0.018, 0.178) for those with no cardiac risk factors vs 0.074 (95% CI 0.011, 0.195) for those with at least one cardiac risk factor, *p* = 0.99 heterogeneity). Unadjusted analysis not presented
Netherlands-Groningen breast cancer study	Roos et al. [20]	Heart	Smoking, BMI, diabetes, hypertension, hyper-cholesterolemia, COPD, pulmonary embolism, chemotherapy, endocrine therapy, history of IHD, CAC	Acute coronary event (myocardial infarction (ICD10 I21-I24), coronary revascularization, death from ischemic heart disease (ICD10 I20-I25))	Incidence	0.17 (0.00 to 0.37)	0.18 (0.00 to 0.39)	ERR adjusted for history of IHD, diabetes, BMI, hypercholesterolemia, hypertension, CAC. A number of simpler models also presented, e.g., as shown here adjusted for history of IHD, diabetes, CAC
Netherlands-Groningen breast cancer study	van den Bogaard et al. [21]	Heart	Smoking, BMI, diabetes, hypertension, hyper-cholesterolemia, chemotherapy, hormonal therapy, trastuzumab, other heart disease	Myocardial infarction (ICD10 I21-24), coronary revascularisation or death from IHD (ICD10 I20-I25) among patients with atherosclerotic plaque in LAD	Incidence	0.116 (-0.079 to 0.353)	0.117 (-0.098 to 0.383)	ERR adjusted for history of IHD, hypertension, hypercholesterolemia, diabetes. Unadjusted ERR are also presented
				Myocardial infarction (ICD10 I21-24), coronary revascularisation or death from IHD (ICD10 I20-I25) among patients without atherosclerotic plaque in LAD		0.153 (-0.017 to 0.353)	0.161 (-0.034 to 0.395)	
Netherlands-NKI-Rotterdam breast cancer case–control study	Jacobse et al. [22]	Heart	Smoking, BMI, hypertension, diabetes, surgery, chemotherapy, endocrine therapy, prior CVD	Myocardial infarction	Incidence	0.064 (0.013 to 0.160)	NA	Although many adjusting variables are available, only ERR for unadjusted analysis is presented. The “dose response relationship for women with and without cardiovascular risk factors at BC diagnosis was similar (*p* > 0.5)”. There are modifications in risk by age at exposure (ERR/Gy age < 45 = 0.242 (95% CI 0.044, 0. 823), age 45–49 = 0.111 (95% CI 0.012, 0.401), age 50–70 = 0.025, 95% CI -0.014, 0.119, heterogeneity *p* = 0.07), and also increased with longer follow-up (ERR/Gy < 10 y = -0.001 (95% CI -0.029, 0. 095), 10–14 y = 0.072 (95% CI -0.008, 0. 323), ≥ 15 y = 0.151, 95% CI 0.029, 0.493, heterogeneity *p* = 0.25)
Netherlands-NKI-Rotterdam breast cancer case–control study	Boekel et al. [23]	Heart	Smoking, BMI, diabetes, hypertension, hyper-cholesterolemia, menopausal status, chemotherapy, endocrine therapy, surgery	Heart failure (CTCAE v3.0, v4.0 grade ≥ 2) – no treatment with anthracyclines	Incidence	0.00 (-0.03 to 0.08)	NA	Although many adjusting variables are available, only ERR for unadjusted analysis is presented. A modifying effect of anthracycline is suggested (but there is no test of significance)
				Heart failure (CTCAE v3.0, v4.0 grade ≥ 2) – treatment with anthracyclines		0.08 (-0.03 to 0.43)	NA	
				Heart failure (CTCAE v3.0, v4.0 grade ≥ 2)		0.01 (-0.02 to 0.10)	NA	
Case–control study nested within ESCaRa breast cancer cohort study	Baaken et al. [24]	Heart	BMI, chemotherapy, endocrine therapy, previous CVD	Myocardial infarction, angina pectoris, congestive heart failure, dysrhythmia, valvular heart disease, or mortality from cardiac infarction (ICD10 I21-I23), chronic IHD (ICD10 I25.0-I25.9), acute IHD (ICD10 I21.0-I24.9), congestive heart failure (ICD10 I50.0-I50.9), angina pectoris (ICD10 I20.0-I20.9), cardiac arrest (ICD10 I46), dysrhythmia/conduction disorder (ICD10 I44.0-I49.9), vitium cordis (ICD10 I34.0-I37.9)	Incidence	-0.01 (-0.06 to 0.05)	-0.01 (-0.06 to 0.05)	Analysis adjusted for chemotherapy, endocrine therapy and BMI. Unadjusted ERR also presented
Severance Hospital breast cancer study	Chung et al. [25]	Heart	Smoking, BMI, diabetes, hypertension, exercise, surgery, chemotherapy, endocrine treatment	Stable angina pectoris, unstable angina, myocardial infarction, IHD, heart failure, atrial fibrillation, coronary revascularisation, death from IHD	Incidence	0.22 (0.13 to 0.30)	0.23 (0.15 to 0.32)	Analysis adjusted for exercise status, BMI, hypertension, diabetes, previous heart disease, anthracycline, anti-HER2 treatment, aromatase inhibitors. Unadjusted analysis also presented
Severance Hospital breast cancer substudy	Kim et al. [26]	Heart	Smoking, BMI, diabetes, hypertension, exercise, anthracycline and other chemotherapy, anti-HER2 treatment, surgery, previous CVD	Acute coronary events (ST-elevation/non-ST-elevation myocardial infarction and unstable angina pectoris)	Incidence	0.03 (0.00 to 0.05)^c**^	0.22 (0.01 to 0.46)^c**^	Analysis adjusted for BMI, laterality, diabetes, smoking, anthracyclines, history of heart disease. Unadjusted analysis also presented
				Heart disease other than acute coronary events		0.20 (0.11, 0.29)^*^	0.13 (0.03 to 0.24)^*^	Analysis adjusted for BMI, laterality, diabetes, smoking, type of surgery, anthracyclines, anti-HER2 treatment, CAC score. Unadjusted analysis also presented
University of Michigan non-small cell lung cancer study	Dess et al. [27]	Heart EQD2	Smoking, diabetes, systolic blood pressure, cholesterol, previous CVD, KPS	CTCAE v4.03 grade ≥ 3 cardiac event	Incidence	0.08 (0.03 to 0.13)	0.07 (0.02 to 0.13)	Analysis adjusted for sex, diabetes, smoking, systolic blood pressure, previous cardiac disease, Framingham risk score. Unadjusted analysis also presented
University of Michigan non-small cell lung cancer study	Xue et al. [28]	Pericardium	Smoking, hypertension, COPD, chemotherapy, previous CVD, KPS	Pericardial effusion	Incidence	0.066 (0.028 to 0.105)	0.050 (0.009 to 0.093)	Analysis adjusted for sex, smoking, hypertension, COPD, chemotherapy, previous CVD, KPS. Unadjusted analysis also presented
Dana Farber/Brigham Women’s Hospital non-small cell lung cancer study	Atkins et al. [29, 30]	Heart	Smoking, BMI, diabetes, hypertension, cholesterol, hyperlipidemia, previous CVD, chemotherapy, statins	Major adverse cardiac events (cardiac death, unstable angina, myocardial infarction, heart failure hospitalization or urgent visit, coronary revascularization)	Incidence/ mortality	0.02 (0.00 to 0.06)	0.03 (0.00 to 0.06)	Adjusted for hypertension, hyperlipidemia, diabetes, previous CVD (stroke, peripheral vascular disease, coronary artery disease, myocardial infarction, congestive heart failure), arrhythmia, valvulopathy, Framingham score, statins. Unadjusted analysis also presented
New Jersey non-small cell lung cancer study	Yegya-Raman et al. [31]	Heart	Smoking, blood pressure, diabetes, chemotherapy, pre-treatment CAD	First symptomatic cardiac event (myocardial infarction, unstable angina, significant arrhythmia, symptomatic pericardial effusion, pericarditis, congestive heart failure)	Incidence	0.058 (0.034 to 0.082)	0.065 (0.038 to 0.093)^d^ 0.059 (0.032 to 0.086)^e^	Two different adjusted models, adjusted for (a) CAD or (b) WHO/ISH stratum, also unadjusted
Chenyang thymoma study	Liao et al. [32]	Heart	Smoking, BMI, diabetes, hypertension, hyperlipidemia, chemotherapy, myasthenia gravis, family history of CVD	CTCAE v4.0 grade ≥ 2 cardiovascular disease	Incidence	0.074 (0.012 to 0.136)^f^	0.084 (0.026 to 0.143)^g^	Adjusted for chemotherapy status, hypertension, hyperlipidemia, diabetes, BMI, family history of CVD. Unadjusted case and control numbers also given
Israeli tinea capitis prevalence study	Sadetzki et al. [33]	Breast	Smoking, BMI, diabetes, hypertension, SES	IHD	Prevalence	NA	7 (1 to 14)^h^	Adjusted for sex, smoking, SES, hypertension, diabetes. Unadjusted analysis not presented for dose response, although analysis of exposure to radiation (yes vs no) suggests that adjustment for these variables made difference of < 2% (all CVD RR = 1.21 unadjusted vs 1.19 adjusted)
		Brain		CeVD		NA	0.20 (0.12 to 0.29)^h^	
		Salivary		Carotid artery stenosis		NA	0.33 (0.04 to 0.71)^h^	
Rochester thymus enlargement study	Adams et al. [34]	Heart	Smoking, dyslipidemia, diabetes, hypertension, family history of myocardial infarction	Coronary heart disease (ICD10 I21-I25, I46)	Incidence	0.08 (-0.01 to 0.20)^***^	-0.03 (-0.07 to 0.10)^***^	Adjusted for sex, diabetes, dyslipidemia, smoking, hypertension. Unadjusted analysis (adjusted for sex) also presented
				Myocardial infarction (ICD10 I21-I24)		-0.05 (-0.13 to 0.08)	-0.06 (-0.16 to 0.06)	
Diagnostically exposed groups								
Canadian and Massachusetts tuberculosis fluoroscopy cohorts	Tran et al. [35]	Lung	Smoking, diabetes, alcohol consumption, antibiotic use, tuberculosis stage	CVD (ICD9 390–459)	Mortality	-0.024 (-0.042 to -0.005)^i^	NA	Main analyses adjust for cohort, sex, smoking, tuberculosis status. Additional analysis for some endpoints adjusts for alcohol consumption, diabetes, antibiotic use in Massachusetts cohort
				CVD (ICD9 390–459) < 0.5 Gy		0.246 (0.036 to 0.469)^i^	0.262 (0.042 to 0.476)^i,j^	
				Ischemic heart disease (ICD9 410–414)		-0.037 (-0.060 to -0.013)^i^	NA	
				Ischemic heart disease (ICD9 410–414) < 0.5 Gy		0.268 (0.003 to 0.552)	0.286 (0.020 to 0.572)^i,j^	
				Cerebrovascular disease (ICD9 430–438)		-0.014 (-0.067 to 0.044)^i^	NA	
				Cerebrovascular disease (ICD9 430–438) < 0.5 Gy		0.441 (-0.119 to 1.090)^i^	0.435 (-0.125 to 1.083)^i,j^	
				Hypertensive heart disease (ICD9 401–405)		-0.035 (-0.152 to 0.153)^i^	NA	
				Hypertensive heart disease (ICD9 401–405) < 0.5 Gy		1.121 (-0.351 to 3.228)^i^	1.147 (-0.333 to 3.266)^i,j^	
				Heart disease apart from hypertensive and IHD (ICD9 390–400, 406–410)		-0.010 (-0.064 to 0.043)^i^	NA	
				Heart disease apart from hypertensive and IHD (ICD9 390–400, 406–410) < 0.5 Gy		-0.226 (-0.679 to 0.307)^i^	-0.240 (-0.691 to 0.291)^i,j^	
				CVD apart from heart and cerebrovascular (ICD9 439–459)		0.055 (-0.028 to 0.164)^i^	NA	
				CVD apart from heart and cerebrovascular (ICD9 439–459) < 0.5 Gy		0.507 (-0.322 to 1.541)^i^	0.511 (-0.320 to 1.547)^i,j^	

*BC* breast cancer, *BMI* body mass index, *CAC* coronary artery calcium, *CAD* coronary artery disease, *CI* confidence interval, *COPD* chronic obstructive pulmonary disease, *CTCAE v* Common Terminology Criteria for Adverse Events version, *CVD* cardiovascular disease, *ERR* excess relative risk, *HER2* human epidermal growth factor receptor 2, *HL* Hodgkin lymphoma; *HRT* hormone replacement therapy, *ICD* International Classification of Diseases, *IHD* ischemic heart disease, *KPS* Karnovsky performance score, *LAD* left anterior descending artery, *MHD* mean heart dose, *MLVD* mean left ventricular dose, *NA* not available, *RR* relative risk, *SES* socioeconomic status, *WHO/ISH* World Health Organization/International Society of Hypertension

^*^ratio of adjusted to unadjusted estimates outside the interval [0.667, 1.5]

^**^ratio of adjusted to unadjusted estimates outside the interval [0.5, 2]

^***^ratio of adjusted to unadjusted estimates negative

^†^ratio of estimate with interaction modifier to that without modifier outside the interval [0.667, 1.5],

^††^ratio of estimate with interaction modifier to that without modifier outside the interval [0.5, 2]

^†††^ratio of estimate with interaction modifier to that without modifier negative

^a^estimate derived by fitting a linear model by (inverse-variance) weighted least squares, applied to the aggregate data provided in Table 3 of Mulrooney et al. [13] and in Table 2 of Shrestha et al. [36]. For the data of Mulrooney et al. [13] (all endpoints except all cardiac disease) average cardiac doses of 0, 7.5, 25, and 45 Gy were assumed for the respective groups with the following specified ranges of cardiac doses: 0, 1–15, 15.1–34.99 Gy, ≥ 35 Gy. For the data of Shrestha et al. [36] average cardiac doses of 0, 5, 15, 25 and 35 Gy were assumed for the respective groups with the following specified ranges of cardiac doses: 0, 0.1–9.9, 10–19.9, 20–29.9 Gy, ≥ 30 Gy, and the central estimates of ERR/Gy given in Figure 5 of Shrestha et al. [36] were used to correct the central estimates of trend

^b^estimate derived by fitting a linear binomial odds model to aggregate numbers of cases and controls, and assuming mean heart doses of 0, 16, 23, 28, 33 Gy for the 0, 1–20, 21–25, 26–30, ≥ 31 Gy mean heart dose groups given in Table 2 of van Nimwegen et al. [18]: see Supplements S1 and S2 of Little et al. [12]

^c^using midpoint estimates for [*D*_*min*_ + 3]/2 and [*D*_*max*_ + 3]/2 Gy (using given minimum and maximum heart doses, *D*_*min*_ and *D*_*max*_) and ln[odds ratio] (and CI) for > 3 Gy vs < 3 Gy from Kim et al. [26]

^d^adjusted using CAD in study of Yegya-Raman et al. [31]

^e^adjusted using WHO/ISH in study of Yegya-Raman et al. [31]

^f^estimate derived by fitting a log-linear binomial odds model to aggregate numbers of cases and controls, and assuming mean heart doses of 5, 15, 25 Gy for the 0–10, 10–20 and 20–30 Gy mean heart dose groups given in Table 4 of Liao et al. [32]: see Supplements S1 and S2 of Little et al. [12]

^g^estimate derived by fitting a log-linear model by (inverse-variance) weighted least squares, applied to the multivariate adjusted ln[OR], and assuming mean heart doses of 5, 15, 25 Gy for the 0–10, 10–20 and 20–30 Gy mean heart dose groups given in Table 4 of Liao et al. [32]

^h^prevalence excess odds ratio per Gy

^i^based on 5-year lagged lung dose

^j^additional adjustment for alcohol consumption, diabetes, antibiotic use

**Table 2:** Unadjusted or adjusted estimated excess relative risks of cardiovascular diseases in the Japanese atomic bomb survivors and in other groups with moderate- or low-dose radiation exposure, with mean dose generally < 0.5 Gy

Study	Reference	Organ used	Variables (other than age, sex, year) available to assess possible confounding	Endpoint	Mortality, incidence or prevalence	Unadjusted (or less adjusted) excess relative risk Gy^−1^ (95% CI)	Fully adjusted excess relative risk Gy^−1^ (95% CI)	Notes
**Japanese atomic bomb survivors**								
Japanese atomic bomb survivors	Shimizu et al. [10] Little et al. [11]	Colon	Smoking, obesity (BMI), diabetes, alcohol intake, education, type of household occupation, city	Heart disease	Mortality	0.122	0.123	Main analyses unadjusted for anything apart from sex, city. Additional analysis adjusting for smoking, alcohol intake, education, type of household occupation, obesity (BMI), diabetes. There were significant modifying effects on CeVD risk of attained age (*p* = 0.04) and age at exposure (*p* = 0.007) (with ERR decreasing with increases in each of these), but no such modifications for heart disease (*p* > 0.2). There are significant modifying effects of sex for heart disease (*p* = 0.022) but not for any other endpoint (*p* > 0.5)
				CeVD		0.081	0.072	
				Other CVD		0.024^**^	0.009^**^	
				CVD (ICD9 390–459)		0.100	0.096	
Japanese atomic bomb survivors	Yamada et al. [9]	Stomach	Smoking, alcohol consumption, city	Hypertension, 1958–1998 (ICD9 401)	Incidence	0.04 (-0.01 to 0.09)^a^	0.05 (-0.01 to 0.10)^a^	All analyses adjusted for city, sex. Additional analyses adjust for smoking and drinking
				Hypertensive heart disease, 1958–1998 (ICD9 402, 404)		0.01 (-0.08 to 0.10)^a***^	-0.01 (-0.09 to 0.09)^a***^	
				IHD, 1958–1998 (ICD9 410–414)		0.04 (-0.06 to 0.14)^a^	0.05 (-0.05 to 0.16)^a^	
				Myocardial infarction, 1964–1998 (ICD9 410)		0.11 (-0.10 to 0.46)^a^	0.12 (-0.16 to 0.60)^a^	
				Occlusion, 1958–1998 (ICD9 433, 434)		0.05 (-0.12 to 0.27)^a^	0.06 (-0.11 to 0.30)^a^	
				Aortic aneurysm, 1958–1998 (ICD9 441, 442)		0.05 (-0.12 to 0.44)^a**^	0.02 (-0.22 to 0.41)^a**^	
				Stroke, 1958–1998 (ICD9 430, 431, 433, 434, 436)		0.06 (-0.08 to 0.23)^a^	0.07 (-0.08 to 0.24)^a^	
Japanese atomic bomb survivors 1958–2014	Takahashi et al. [37]	Skin	Smoking, BMI, diabetes, blood pressure, total + LDL and HDL cholesterol, triglycerides, dyslipidemia, high sensitivity CRP, white blood cell count, GFR, city	Peripheral artery disease	Prevalence	-0.11 (-0.39, 0.30)^*^	-0.17 (-0.43 to 0.22)^*^	Analyses adjusted for sex, smoking, CRP, GFR, hypertension, diabetes, dyslipidemia, also unadjusted
**Occupational studies**								
International Agency for Research on Cancer15-country nuclear worker study	Vrijheid et al. [38]	Colon	Employer/facility, SES	CVD (ICD10 I00-I99, J60-J69, O88.2, R00-R02, R57)	Mortality	NA	0.09 (-0.43 to 0.70)^c^	Analyses adjusted for employer/facility, sex, SES. No unadjusted analyses presented. There is no significant variation of ERR by gender, age at exposure or attained age for all CVD (*p* ≥ 0.1), although there is highly significant increase in ERR with increasing duration of employment (*p* = 0.01)
				IHD (ICD10 I20-I25)		NA	-0.01 (-0.59 to 0.69)^c^	
				Heart failure (ICD10 I50)		NA	-0.03 (< 0 to 4.91)^c^	
				Deep vein thrombosis and pulmonary embolism (ICD10 I26, I80, I82, O88.2)		NA	-0.95 (-1.00 to 9.09)^c, l^	
				CeVD (ICD10 I60-I69)		NA	0.88 (-0.67 to 3.16)^c^	
				All other CVD (ICD10 R00-R02, R57, I00-I99 excluding I20-26, I50, I60-69, I80, I82)			0.29 (< 0 to 2.40)^c^	
International Nuclear Workers Study (INWORKS)	Gillies et al. [39]	Film badge (H_p_(10))	Employer/facility, SES	CVD (ICD10 I00-I99)	Mortality	NA	0.22 (0.08 to 0.37)^m^	Analyses adjusted for employer/facility, sex, SES. No unadjusted analyses presented. There are significant differences in ERR by sex for CVD (*p* = 0.005) and for IHD (*p* = 0.004), but not for CeVD (*p* > 0.5). There are no significant differences in ERR by attained age or duration of employment (*p* > 0.5 for all endpoints)
				CVD (ICD10 I00-I99) males		NA	0.20 (0.07 to 0.36)^m^	
				CVD (ICD10 I00-I99) females		NA	4.22 (1.72 to 7.21)^m ††^	
				CVD (ICD10 I00-I99) duration of employment < 10 y		NA	0.27 (-0.31 to 0.90)^m^	
				CVD (ICD10 I00-I99) duration of employment 10–19 y		NA	0.15 (-0.14 to 0.47)^m^	
				CVD (ICD10 I00-I99) duration of employment 20–29 y		NA	0.27 (0.06 to 0.49)^m^	
				CVD (ICD10 I00-I99) duration of employment ≥ 30 y		NA	0.19 (-0.03 to 0.42)^m^	
				CVD (ICD10 I00-I99) age < 60		NA	0.59 (0.15 to 1.08)^m^	
				CVD (ICD10 I00-I99) age 60–69		NA	0.05 (-0.20 to 0.31)^m^	
				CVD (ICD10 I00-I99) age ≥ 70		NA	0.23 (0.06 to 0.41)^m^	
				IHD (I20-I25)		NA	0.18 (0.004 to 0.36)^m^	
				IHD (I20-I25) males		NA	0.16 (-0.01 to 0.34)^m^	
				IHD (I20-I25) females		NA	6.17 (2.44 to 10.92)^m ††^	
				IHD (I20-I25) duration of employment < 10 y		NA	0.04 (-0.67 to 0.83)^m^	
				IHD (I20-I25) duration of employment 10–19 y		NA	0.17 (-0.19 to 0.57)^m^	
				IHD (I20-I25) duration of employment 20–29 y		NA	0.16 (-0.09 to 0.42)^m^	
				IHD (I20-I25) duration of employment ≥ 30 y		NA	0.21 (-0.06 to 0.51)^m^	
				IHD (I20-I25) age < 60		NA	0.64 (0.14 to 1.20)^m^	
				IHD (I20-I25) age 60–69		NA	-0.02 (-0.30 to 0.29)^m^	
				IHD (I20-I25) age ≥ 70		NA	0.18 (-0.04 to 0.41)^m^	
				Acute myocardial infarction (I21)		NA	0.26 (0.03 to 0.51)^m^	
				Chronic ischemic heart disease (I25)		NA	0.07 (-0.19 to 0.36)^m^	
				CeVD (I60-I69)		NA	0.50 (0.12 to 0.94)^m^	
				CeVD (ICD10 I60-I69) males		NA	0.48 (0.10 to 0.91)^m^	
				CeVD (ICD10 I60-I69) females		NA	2.67 (< 0 to 9.79)^m^	
				CeVD (I60-I69) duration of employment < 10 y		NA	1.01 (-0.58 to 2.90)^m^	
				CeVD (I60-I69) duration of employment 10–19 y		NA	0.35 (-0.34 to 1.19)^m^	
				CeVD (I60-I69) duration of employment 20–29 y		NA	0.70 (0.16 to 1.35)^m^	
				CeVD (I60-I69) duration of employment ≥ 30 y		NA	0.21 (-0.36 to 0.94)^m^	
				CeVD (ICD10 I60-I69) age < 60		NA	0.06 (< 0 to 1.99)^m^	
				CeVD (ICD10 I60-I69) age 60–69		NA	-0.18 (< 0 to 0.72)^m^	
				CeVD (ICD10 I60-I69) age ≥ 70		NA	0.66 (0.22 to 1.17)^m^	
Mayak workers IHD	Azizova et al. [40, 41]	External gamma	Smoking, BMI, hypertension alcohol consumption, migration status	IHD (ICD9 410–414) < 4 Gy	Incidence	0.16 (0.10 to 0.23)^b^	0.14 (0.08 to 0.21)^b^	Smoking, alcohol consumption, hypertension, BMI, sex adjusted for. For the mortality data migration status also adjusted for, but there was no adjustment for hypertension. The authors note that in the morbidity data: “The exclusion of the smoking and alcohol consumption adjustments had no effect on the risk estimate for the full data set, but the risk for the dose-restricted data set became significant. Additional adjustment for hypertension did not change the result while the BMI adjustment caused the increase of the risk estimates for ... both data sets and the risk became significant.”; while in the mortality data: “In male residents, exclusion and inclusion of additional adjustments for non-radiation factors in the model changed the magnitude of the risk and made confidence intervals wider, but not the significance, except the adjustment for BMI in the IHD analysis, which increased the ERR/Gy by 116% and produced a significant result … meanwhile the [ischemic stroke] mortality risk for male residents remained significant.”
				IHD (ICD9 410–414) < 4 Gy		0.17 (0.11 to 0.25)^c^	0.15 (0.09 to 0.22)^c^	
				IHD (ICD9 410–414) < 4 Gy		0.17 (0.11 to 0.25)^d^	0.17 (0.10 to 0.25)^d^	
				IHD (ICD9 410–414) < 4 Gy	Mortality	0.08 (0.01 to 0.17)^b^	0.07 (< 0 to 0.16)^b^	
				IHD (ICD9 410–414) < 4 Gy		0.08 (0.01 to 0.17)^c^	0.07 (> 0 to 0.16)^c^	
				IHD (ICD9 410–414) < 4 Gy		0.08 (0.01 to 0.17)^d^	0.07 (< 0 to 0.16)^d^	
		Liver external gamma	Smoking, BMI, diabetes, alcohol consumption	IHD (ICD9 410–414)	Mortality	0.07 (> 0 to 0.14)^c*^	0.04 (-0.02 to 0.11)^c*^	
Mayak workers CeVD	Azizova et al. [41–43]	External gamma	Smoking, BMI, hypertension, alcohol consumption	CeVD (ICD9 430–438)	Incidence	0.46 (0.35 to 0.53)^b^	0.46 (0.37 to 0.56)^b^	Adjusted for smoking, alcohol consumption, hypertension, BMI, sex. The authors noted that “adjusting for additional non-radiation factors (hypertension, body mass index, duration of employment, smoking index) or liver dose from internal alpha-particle radiation did not significantly change this [ERR trend] result.” Partially unadjusted analysis (adding BMI or hypertension to analysis already adjusted for other factors, or dropping smoking and alcohol consumption) also presented, and made < 5% difference
				CeVD (ICD9 430–438)		0.49 (0.37 to 0.57)^c^	0.49 (0.39 to 0.60)^c^	
				CeVD (ICD9 430–438)		0.53 (0.41 to 0.66)^d^	0.53 (0.43 to 0.65)^d^	
		Liver external gamma	Smoking, BMI, diabetes, alcohol consumption	CeVD (ICD9 430–438)		0.42 (0.34 to 0.51)^c, e^	0.39 (0.31 to 0.48)^c^	Analysis adjusted for sex, smoking, BMI, diabetes, alcohol consumption. Partially unadjusted analysis (dropping smoking and alcohol consumption) also presented
		External gamma	Smoking, BMI, hypertension, alcohol consumption, migration status	CeVD (ICD9 430–438)	Mortality	0.05 (-0.03 to 0.15)^b^	0.05 (-0.04 to 0.16)^b^	Analysis adjusted for sex, smoking, BMI, hypertension, alcohol consumption. Partially unadjusted analysis (adding BMI or hypertension to unlagged analysis already adjusted for other factors, or dropping smoking and alcohol consumption) also presented, and made little difference (ERR/Gy in range 0.05—0.08)
				CeVD (ICD9 430–438)		0.06 (-0.03 to 0.16)^c^	0.05 (-0.03 to 0.16)^c^	
				CeVD (ICD9 430–438)		0.07 (-0.02 to 0.17)^d^	0.06 (-0.03 to 0.18)^d^	
Mayak workers all cardiovascular disease	Azizova et al. [41, 44]	External gamma	Smoking, BMI, hypertension, alcohol consumption	CVD (ICD9 390–459) < 4 Gy	Mortality	0.08 (0.03 to 0.15)^b^	0.08 (0.02 to 0.14)^b^	Adjusted for smoking, alcohol consumption, BMI, hypertension, sex. The authors noted that “The results observed for both the full dataset and the dose-restricted one remained unchanged after … exclusion of adjustments for smoking and alcohol consumption and inclusion of adjustments for additional non-radiation factors.”
						0.09 (0.03 to 0.15)^c^	0.08 (0.02 to 0.15)^c^	
						0.09 (0.03 to 0.16)^d^	0.09 (0.03 to 0.16)^d^	
		Liver external gamma	Smoking, BMI, diabetes, alcohol consumption, hypertension	CVD (ICD9 390–459)		0.06 (0.01 to 0.12)^c*^	0.03 (-0.01 to 0.09)^c*^	Analysis in which various adjustment factors (smoking, BMI, diabetes, hypertension) were individually added as adjustments made little difference for component CVD endpoints
Mayak workers lower extremity arterial disease	Azizova et al. [45]	External gamma	Smoking, BMI, hypertension, alcohol consumption, diabetes	Lower extremity arterial disease (ICD9 440.2)	Incidence	0.34 (0.16 to 0.58)^b^	0.30 (0.13 to 0.53)^b^	Adjusted for sex, smoking, BMI, hypertension, alcohol consumption, diabetes. The authors note that “ERR/Gy … slightly increases with the exclusion of adjustments for smoking and alcohol consumption. Inclusion of adjustments for hypertension, diabetes mellitus and duration of employment shows no change in the obtained result. On the contrary, inclusion of additional adjustments for BMI and smoking index (rather than smoking status) results in a slight decrease of ERR/Gy.”
				Lower extremity arterial disease (ICD9 440.2)		0.31 (0.15 to 0.56)^c^	0.28 (0.12 to 0.50)^c^	
				Lower extremity arterial disease (ICD9 440.2)		0.36 (0.18 to 0.62)^d^	0.32 (0.14 to 0.57)^d^	
Mayak workers hypertension	Azizova et al. [46]	Liver external gamma	Smoking, BMI, alcohol consumption	Hypertension (ICD9 401–404)	Incidence	0.14 (0.08 to 0.20)^b^	0.15 (0.09 to 0.21)^b^	Adjusted for sex, smoking, BMI, alcohol consumption. The authors note that: “The hypertension risk estimates … decreased after exclusion of adjustments for smoking status and alcohol consumption and inclusion of an adjustment for the baseline blood pressure level. The hypertension incidence risk was revealed to increase with inclusion into stratification of adjustments for additional non-radiation factors (body mass index and duration of employment), but the smoking index adjustment did not modify the risk estimate.”
						0.16 (0.10 to 0.22)^c^	0.17 (0.11 to 0.24)^c^	
						0.17 (0.11 to 0.24)^d^	0.19 (0.12 to 0.26)^d^	
French nuclear fuel cycle workers	Bouet et al. [47]	External gamma	Smoking, BMI, glycemic level, hypertension, SES	CVD (ICD10 I00-I99, G45-G46) (subgroup 2, adjusted for SES, blood pressure, BMI, smoking status, glycemic level)	Mortality	-3.5 (NA to 15.0)^f**^	-0.1 (NA to 48.4)^**^	Adjusted for smoking, BMI, glycemic level, hypertension, SES
				IHD (ICD10 I20-I25) (subgroup 1)		-4.0 (NA to 34.7)	NA	Adjusted for sex, SES. Fully adjusted analysis not presented
				CeVD (ICD10 I60-I69 + G45 (exc G45.3, G45.4) + G46) (subgroup 1)		-0.3 (NA to 61.4)	NA	
French uranium miners case–control study	Drubay et al. [48]	External gamma	Smoking, BMI, diabetes, hypertension, hyper-cholesterolemia, hyper-triglyceridemia, resting heart rate, chronic kidney disease (via GFR), hyperuricemia, gamma glutamyl transpeptidase	CVD (ICD10 I00-I99)	Mortality	0.4 (-1.8 to 3.0) ^***^ 0.4 (-1.6 to 2.9)^g^	-0.7 (-3.2 to 2.9) ^***^	Adjusted for smoking, BMI, diabetes, hypertension, hyper-cholesterolemia, hyper-triglyceridemia, resting heart rate, chronic kidney disease (via GFR), hyperuricemia, gamma glutamyl transpeptidase. Adjusted and unadjusted ERR presented, as well as counter-matched analysis
				IHD (ICD10 I20-I25)		-1.1 (-4.0 to 3.2) ^**^ -1.0 (-3.9 to 3.3)^g^	-2.6 (-6.3 to 5.1) ^**^	
				CeVD (ICD10 I60-I69)		3.7 (-0.9 to 10.6) ^*^ 2.4 (-0.6 to 11.4)^g^	1.9 (-3.5 to 11.8) ^*^	
US nuclear power workers	Boice et al. [49]	Heart	SES, worker mobility, duration of monitoring	IHD (ICD9 410–414)	Mortality	NA	-0.1 (-0.6 to 0.4)	Sex, SES were adjusted for. Unadjusted analyses were not presented, although analysis of leukemia suggested that adding worker mobility or duration of monitoring as adjustments, or removing sex or SES from model had little effect
NASA astronauts	Elgart et al. [50]	Effective dose	Medical radiation dose	CVD (IHD, CeVD)	Mortality	-116.4 (-462.1 to 16.1)^h^ -120.1 (-474.0 to 16.1)^i^	-123.5 (-491.6 to 16.9)	Adjusted for age at entrance, age at exit and medical diagnostic dose. Analysis unadjusted for medical diagnostic dose, age at entrance also given
				IHD		-60.0 (-382.9 to 34.3)^h^ -60.7 (-389.3 to 34.3)^i^	-62.7 (-411.8 to 32.6)	
				CeVD		-580.7 (-600.3 to 22.9)^h^ -616.1 (-636.8 to > 0)^i^	-501.5 (-925.1 to 32.9)	
Korean medical diagnostic workers	Cha et al. [51]	Heart	Smoking, BMI, blood glucose, systolic + diastolic blood pressure, total + low-density LDL + high-density LDL cholesterol, alcohol intake	CVD (ICD10 I00-I83, I85-I99)	Incidence	1.5 (-3.9 to 8.0) ^**^	0.6 (-4.6 to 6.8) ^**^	Adjusted for sex, smoking, alcohol intake, BMI, systolic + diastolic blood pressure, total + LDL + HDL cholesterol, blood glucose. Unadjusted analyses also presented, adjusted only for sex. The modifications in ERR (adjusted for age, sex, birth year) were significant for sex (*p* = 0.03), attained age (*p* = 0.004), year starting work (*p* = 0.003) and total years worked (*p* = 0.007). There were similar generally significant modifications in ERR for hypertension, and CVD excluding CeVD and others, but not for IHD, CeVD and other CVD
				CVD (ICD10 I00-I83, I85-I99) male		-0.7 (-7.6 to 7.6) ^†††^	NA	
				CVD (ICD10 I00-I83, I85-I99) female		42.1 (3.0 to 99.2) ^†††^	NA	
				CVD (ICD10 I00-I83, I85-I99) age < 50 y		9.8 (0.2 to 21.0) ^†††^	NA	
				CVD (ICD10 I00-I83, I85-I99) age ≥ 50 y		-5.1 (-12.0 to 3.5) ^†††^	NA	
				CVD (ICD10 I00-I83, I85-I99) birth < 1970		-1.8 (-8.8 to 6.7) ^†††^	NA	
				CVD (ICD10 I00-I83, I85-I99) birth ≥ 1970		14.5 (-0.1 to 31.7) ^†††^	NA	
				CVD (ICD10 I00-I83, I85-I99) year started work < 2000		-0.6 (-7.3 to 7.6) ^†††^	NA	
				CVD (ICD10 I00-I83, I85-I99) year started work ≥ 2000		44.5 (13.4 to 82.2) ^†††^	NA	
				CVD (ICD10 I00-I83, I85-I99) years worked < 10 y		45.8 (5.3 to 97.1) ^†††^	NA	
				CVD (ICD10 I00-I83, I85-I99) years worked 10–19 y		11.1 (-0.8 to 25.2) ^†††^	NA	
				CVD (ICD10 I00-I83, I85-I99) years worked ≥ 20 y		-3.6 (-10.6 to 4.9) ^†††^	NA	
				Hypertension (ICD10 I10-I15)		0.3 (-6.9 to 9.6) ^***^	-1.8 (-8.3 to 6.8) ^***^	
				IHD (ICD10 I20-I25)		8.7 (-7.8 to 38.2) ^*^	5.3 (< -11.3 to 32.4) ^*^	
				CeVD (ICD10 I60-I69)		12.2 (< -12.2 to 60.0)	12.0 (< -12.3 to 58.8)	
				Other CVD (ICD10 I70-I83, I85-I99)		-0.4 (< -11.9 to 15.0)^*^	-0.2 (< -11.7 to 15.5) ^*^	
				CVD excluding CeVD and others (ICD10 I53-I59, I70-I83, I85-I99)		1.3 (-5.0 to 9.0) ^***^	-0.5 (-6.3 to 6.7) ^***^	
Korean radiation workers prevalence study	Park et al. [52]	Film badge (H_p_(10))	Smoking, BMI, diabetes, alcohol consumption, hyperlipidemia, cataracts, hepatitis, diseases of thyroid, musculoskeletal and respiratory systems, occupation, duration of employment, regular exercise, night shift work	CVD (ICD10 I00-I99)	Prevalence	8 (6 to 9)^j**^	0 (-2 to 2)^j**^	Adjusted for sex, occupation, duration of employment, smoking, alcohol consumption, regular exercise, BMI, night shift work. Unadjusted OR also presented
**Environmental studies**								
Semipalatinsk nuclear test hypertension study	Markabayeva et al. [53]	Effective dose	Smoking, BMI, total cholesterol, hypercholesterolemia, alcohol consumption	Essential hypertension (ICD10 I10)	Prevalence	3.528 (-3.188 to 10.245)^k**^	10.325 (-6.142 to 26.793)^k**^	Adjusted for BMI, total cholesterol, smoking, alcohol, also adjusted only for age, sex

*BMI* body mass index, *CI* confidence interval, *CRP* C-reactive protein, *CeVD* cerebrovascular disease, *CVD* cardiovascular disease, *ICD* International Classification of Diseases, *ERR* excess relative risk, *GFR* glomerular filtration rate, *HDL* high density lipoprotein, *H*_*p*_*(10)* personal dose equivalent at 10 mm depth, *LDL* low density lipoprotein, *OR* odds ratio, *SES* socioeconomic status

^*^ratio of adjusted to unadjusted estimates outside the interval [0.667, 1.5]

^**^ratio of adjusted to unadjusted estimates outside the interval [0.5, 2]

^***^ratio of adjusted to unadjusted estimates negative

^†^ratio of estimate with interaction modifier to that without modifier outside the interval [0.667, 1.5]

^††^ratio of estimate with interaction modifier to that without modifier outside the interval [0.5, 2]

^†††^ratio of estimate with interaction modifier to that without modifier negative

^a^analysis derived from Table 3 of Yamada et al. [9] with smoking and drinking in the stratification

^b^assuming a lag period of 5 years

^c^assuming a lag period of 10 years

^d^assuming a lag period of 15 years

^e^dropping smoking and alcohol consumption from adjustment

^f^adjusting for sex, SES

^g^based on counter-matched analysis

^h^adjusting for age at exit

^i^adjusting for age at exit + entrance, medical diagnostic dose

^j^prevalence excess odds ratio per Gy

^k^estimate derived by fitting a linear model by (inverse-variance) weighted least squares, applied to the adjusted odds ratio (OR) provided in Table 2 of Markabayeva et al. [53]. Median cardiac doses of 0.009, 0.041, 0.070, and 0.326 Sv were assumed for the respective groups with the following specified ranges of effective doses: < 20, 20–59, 60–185, > 185 mSv, as given by Markabayeva et al. [53]

^l^estimate derived from log-linear model, evaluated at 1 Sv

^m^90% CI

### Methods to evaluate the effects of confounding and effect modifying variables

The methods employed to assess the effects of potential confounding variable are comparison of the fitted ERR unadjusted for the potential confounding variable, $ERR_{unadj}$, and the ERR $ERR_{adj\;\lbrack V\rbrack}$ adjusted for the confounding variable $V$. We categorized those estimates in which adjustment for potential confounders resulted in changes of the following magnitudes:

- more than 50% difference, i.e., with ratio of estimates outside the interval [0.667, 1.5] – labelled *
- more than 100% difference, i.e., with ratio of estimates outside the interval [0.5, 2.0] – labelled **
- estimates with different signs, i.e. one positive, the other negative, labelled ***

Likewise any variable whose interaction with radiation dose resulted in the following degree of change in the ERR was labelled as follows:

- more than 50% difference, i.e., with ratio of estimates (with/without modification) outside the interval [0.667, 1.5] and the heterogeneity was statistically significant (*p* < 0.05) – labelled †
- more than 100% difference, i.e., with ratio of estimates (with/without modification) outside the interval [0.5, 2.0] and the heterogeneity was statistically significant (*p* < 0.05) – labelled ††
- estimates with different signs, i.e. one (with modification) positive, the other (without modification) negative or vice versa and the heterogeneity was statistically significant (*p* < 0.05), labelled †††

We specifically highlight the most serious discrepancies of both sorts (***, †††) in the text below. We describe these as *potential confounders* and *potential effect modifiers*, respectively. Table 3 reports those studies and results (taken from Tables 1, 2) in which one of these six categories of potentially confounding or modifying effects is observed. Table 4 separately reports effects of variation of latency.

**Table 3:** Unadjusted or adjusted estimated excess relative risk of cardiovascular diseases in various groups in which there is pronounced effect of adjustment for potential confounder variables, or significant variation by modifying factors. All analyses adjust for age

Study	Reference	Organ used	Variables (other than age, sex, year) available to assess possible confounding	Endpoint	Mortality, incidence or prevalence	Unadjusted (or less adjusted) excess relative risk Gy^−1^ (95% CI)	Fully adjusted excess relative risk Gy^−1^ (95% CI)	Notes
**Therapeutically treated groups**								
Rochester thymus enlargement study	Adams et al. [34]	Heart	Smoking, dyslipidemia, diabetes, hypertension, family history of myocardial infarction	Coronary heart disease (ICD10 I21-I25, I46)	Incidence	0.08 (-0.01 to 0.20)^***^	-0.03 (-0.07 to 0.10)^***^	Adjusted for sex, diabetes, dyslipidemia, smoking, hypertension. Unadjusted analysis (adjusted for sex) also presented
**Japanese atomic bomb survivors**								
Japanese atomic bomb survivors	Shimizu et al. [10] Little et al. [11]	Colon	Smoking, obesity (BMI), diabetes, alcohol intake, education, type of household occupation, city	CeVD	Mortality	0.081	0.072	Main analyses unadjusted for anything apart from sex, city. Additional analysis adjusting for smoking, alcohol intake, education, type of household occupation, obesity (BMI), diabetes. There were significant modifying effects on CeVD risk of attained age (*p* = 0.04) and age at exposure (*p* = 0.007) (with ERR decreasing with increases in each of these), but no such modifications for heart disease (*p* > 0.2). There are significant modifying effects of sex for heart disease (*p* = 0.022) but not for any other endpoint (*p* > 0.5)
				Other CVD		0.024^**^	0.009^**^	
Japanese atomic bomb survivors	Yamada et al. [9]	Stomach	Smoking, alcohol consumption, city	Hypertensive heart disease, 1958–1998 (ICD9 402, 404)	Incidence	0.01 (-0.08 to 0.10)^a***^	-0.01 (-0.09 to 0.09)^a***^	All analyses adjusted for city, sex. Additional analyses adjust for smoking and drinking
				Aortic aneurysm, 1958–1998 (ICD9 441, 442)		0.05 (-0.12 to 0.44)^a**^	0.02 (-0.22 to 0.41)^a**^	
Japanese atomic bomb survivors 1958–2014	Takahashi et al. [37]	Skin	Smoking, BMI, diabetes, blood pressure, total + LDL and HDL cholesterol, triglycerides, dyslipidemia, high sensitivity CRP, white blood cell count, GFR, city	Peripheral artery disease	Prevalence	-0.11 (-0.39, 0.30)^*^	-0.17 (-0.43 to 0.22)^*^	Analyses adjusted for sex, smoking, CRP, GFR, hypertension, diabetes, dyslipidemia, also unadjusted
**Occupational studies**								
International Nuclear Workers Study (INWORKS)	Gillies et al. [39]	Film badge (H_p_(10))	Employer/facility, SES	CVD (ICD10 I00-I99) females	Mortality	NA	4.22 (1.72 to 7.21)^b^	Analyses adjusted for employer/facility, sex, SES. No unadjusted analyses presented. There are significant differences in ERR by sex for CVD (*p* = 0.005) and for IHD (*p* = 0.004), but not for CeVD (*p* > 0.5). There are no significant differences in ERR by attained age or duration of employment (*p* > 0.5 for all endpoints)
				IHD (I20-I25) females		NA	6.17 (2.44 to 10.92)^b^	
Mayak workers	Azizova et al. [41]	Liver external gamma	Smoking, BMI, diabetes, alcohol consumption	IHD (ICD9 410–414)	Mortality	0.07 (> 0 to 0.14)^c*^	0.04 (-0.02 to 0.11)^c*^	Smoking, alcohol consumption, BMI, sex, migration status adjusted for. The authors note that: “In male residents, exclusion and inclusion of additional adjustments for non-radiation factors in the model changed the magnitude of the risk and made confidence intervals wider, but not the significance, except the adjustment for BMI in the IHD analysis, which increased the ERR/Gy by 116% and produced a significant result … meanwhile the [ischemic stroke] mortality risk for male residents remained significant.”
				CVD (ICD9 390–459)		0.06 (0.01 to 0.12)^c*^	0.03 (-0.01 to 0.09)^c*^	Analysis in which various adjustment factors (smoking, BMI, diabetes, hypertension) were individually added as adjustments made little difference for component CVD endpoints
French nuclear fuel cycle workers	Bouet et al. [47]	External gamma	Smoking, BMI, glycemic level, hypertension, SES	CVD (ICD10 I00-I99, G45-G46) (subgroup 2, adjusted for SES, blood pressure, BMI, smoking status, glycemic level)	Mortality	-3.5 (NA to 15.0)^d**^	-0.1 (NA to 48.4)^**^	Adjusted for smoking, BMI, glycemic level, hypertension, SES
French uranium miners case–control study	Drubay et al. [48]	External gamma	Smoking, BMI, diabetes, hypertension, hyper-cholesterolemia, hyper-triglyceridemia, resting heart rate, chronic kidney disease (via GFR), hyperuricemia, gamma glutamyl transpeptidase	CVD (ICD10 I00-I99)	Mortality	0.4 (-1.8 to 3.0) ^***^ 0.4 (-1.6 to 2.9)^e^	-0.7 (-3.2 to 2.9) ^***^	Adjusted for smoking, BMI, diabetes, hypertension, hyper-cholesterolemia, hyper-triglyceridemia, resting heart rate, chronic kidney disease (via GFR), hyperuricemia, gamma glutamyl transpeptidase. Adusted and unadjusted ERR presented, as well as counter-matched analysis
				IHD (ICD10 I20-I25)		-1.1 (-4.0 to 3.2) ^**^ -1.0 (-3.9 to 3.3)^e^	-2.6 (-6.3 to 5.1) ^**^	
				CeVD (ICD10 I60-I69)		3.7 (-0.9 to 10.6) ^*^ 2.4 (-0.6 to 11.4)^e^	1.9 (-3.5 to 11.8) ^*^	
Korean medical diagnostic workers	Cha et al. [51]	Heart	Smoking, BMI, blood glucose, systolic + diastolic blood pressure, total + low-density LDL + high-density LDL cholesterol, alcohol intake	CVD (ICD10 I00-I83, I85-I99)	Incidence	1.5 (-3.9 to 8.0) ^**^	0.6 (-4.6 to 6.8) ^**^	Adjusted for sex, smoking, alcohol intake, BMI, systolic + diastolic blood pressure, total + LDL + HDL cholesterol, blood glucose. Unadjusted analyses also presented, adjusted only for sex. The modifications in ERR (adjusted for age, sex, birth year) were significant for sex (*p* = 0.03), attained age (*p* = 0.004), year starting work (*p* = 0.003) and total years worked (*p* = 0.007). There were similar generally significant modifications in ERR for hypertension, and CVD excluding CeVD and others, but not for IHD, CeVD and other CVD
				CVD (ICD10 I00-I83, I85-I99) male		-0.7 (-7.6 to 7.6) ^†††^	NA	
				CVD (ICD10 I00-I83, I85-I99) female		42.1 (3.0 to 99.2) ^†††^	NA	
				CVD (ICD10 I00-I83, I85-I99) age < 50 y		9.8 (0.2 to 21.0) ^†††^	NA	
				CVD (ICD10 I00-I83, I85-I99) age ≥ 50 y		-5.1 (-12.0 to 3.5) ^†††^	NA	
				CVD (ICD10 I00-I83, I85-I99) birth < 1970		-1.8 (-8.8 to 6.7) ^†††^	NA	
				CVD (ICD10 I00-I83, I85-I99) birth ≥ 1970		14.5 (-0.1 to 31.7) ^†††^	NA	
				CVD (ICD10 I00-I83, I85-I99) year started work < 2000		-0.6 (-7.3 to 7.6) ^†††^	NA	
				CVD (ICD10 I00-I83, I85-I99) year started work ≥ 2000		44.5 (13.4 to 82.2) ^†††^	NA	
				CVD (ICD10 I00-I83, I85-I99) years worked < 10 y		45.8 (5.3 to 97.1) ^†††^	NA	
				CVD (ICD10 I00-I83, I85-I99) years worked 10–19 y		11.1 (-0.8 to 25.2) ^†††^	NA	
				CVD (ICD10 I00-I83, I85-I99) years worked ≥ 20 y		-3.6 (-10.6 to 4.9) ^†††^	NA	
				Hypertension (ICD10 I10-I15)		0.3 (-6.9 to 9.6) ^***^	-1.8 (-8.3 to 6.8) ^***^	
				IHD (ICD10 I20-I25)		8.7 (-7.8 to 38.2) ^*^	5.3 (< -11.3 to 32.4) ^*^	
				Other CVD (ICD10 I70-I83, I85-I99)		-0.4 (< -11.9 to 15.0)^*^	-0.2 (< -11.7 to 15.5) ^*^	
				CVD excluding CeVD and others (ICD10 I53-I59, I70-I83, I85-I99)		1.3 (-5.0 to 9.0) ^***^	-0.5 (-6.3 to 6.7) ^***^	
Korean radiation workers prevalence study	Park et al. [52]	Film badge (H_p_(10))	Smoking, BMI, diabetes, alcohol consumption, hyperlipidemia, cataracts, hepatitis, diseases of thyroid, musculoskeletal and respiratory systems, occupation, duration of employment, regular exercise, night shift work	CVD (ICD10 I00-I99)	Prevalence	8 (6 to 9)^f**^	0 (-2 to 2)^f**^	Adjusted for sex, occupation, duration of employment, smoking, alcohol consumption, regular exercise, BMI, night shift work. Unadjusted OR also presented
**Environmental studies**								
Semipalatinsk nuclear test hypertension study	Markabayeva et al. [53]	Effective dose	Smoking, BMI, total cholesterol, hypercholesterolemia, alcohol consumption	Essential hypertension (ICD10 I10)	Prevalence	3.528 (-3.188 to 10.245)^g**^	10.325 (-6.142 to 26.793)^g**^	Adjusted for BMI, total cholesterol, smoking, alcohol, also adjusted only for age, sex

*BMI* body mass index, *CI* confidence interval, *CRP* C-reactive protein, *CeVD* cerebrovascular disease, *CVD* cardiovascular disease, *ICD* International Classification of Diseases, *ERR* excess relative risk, *GFR* glomerular filtration rate, *HDL* high density lipoprotein, *H*_*p*_*(10)* personal dose equivalent at 10 mm depth, *LDL* low density lipoprotein, *OR* odds ratio, *SES* socioeconomic status

^*^ratio of adjusted to unadjusted estimates outside the interval [0.667, 1.5]

^**^ratio of adjusted to unadjusted estimates outside the interval [0.5, 2]

^***^ratio of adjusted to unadjusted estimates negative

^†^ratio of estimate with interaction modifier to that without modifier outside the interval [0.667, 1.5]

^††^ratio of estimate with interaction modifier to that without modifier outside the interval [0.5, 2]

^†††^ratio of estimate with interaction modifier to that without modifier negative

^a^analysis derived from Table 3 of Yamada et al. [9] with smoking and drinking in the stratification

^b^90% CI

^c^assuming a lag period of 10 years

^d^adjusting for sex, SES

^e^based on counter-matched analysis

^f^prevalence excess odds ratio per Gy

^g^estimate derived by fitting a linear model by (inverse-variance) weighted least squares, applied to the adjusted odds ratio (OR) provided in Table 2 of Markabayeva et al. [53]. Median cardiac doses of 0.009, 0.041, 0.070, and 0.326 Sv were assumed for the respective groups with the following specified ranges of effective doses: < 20, 20–59, 60–185, > 185 mSv, as given by Markabayeva et al. [53]

**Table 4:** Variation with latency of estimated excess relative risks of cardiovascular diseases in occupationally and environmentally exposed groups

Cohort/Study	Reference	Average Organ Dose (Gy or Sv) mean/ median (range)	Organ used	Variables (other than age, sex, year) available to assess possible confounding	Persons (person years of follow-up)	Deaths/ cases	Mortality or incidence	Lag period (years)	Endpoint	Excess relative risk Gy^−1^ (95% CI)
**Occupational studies**										
Mayak workers IHD	Azizova et al. [40, 41]	0.51 (0 to > 4.5)^a^	External gamma	Smoking, BMI, hypertension alcohol consumption	22,377 (447,281)	7225	Incidence	5	IHD (ICD9 410–414) < 4 Gy	0.14 (0.08 to 0.21)
								10	IHD (ICD9 410–414) < 4 Gy	0.15 (0.09 to 0.22)
								15	IHD (ICD9 410–414) < 4 Gy	0.17 (0.10 to 0.25)
					22,377 (836,048)	2848	Mortality	5	IHD (ICD9 410–414) < 4 Gy	0.07 (< 0 to 0.16)
								10	IHD (ICD9 410–414) < 4 Gy	0.07 (> 0 to 0.16)
								15	IHD (ICD9 410–414) < 4 Gy	0.07 (< 0 to 0.16)
		0.43 (< 0.1 to > 3.0)	Liver external gamma	Smoking, BMI, diabetes, alcohol consumption	22,377 (890,132)	3481		10	IHD (ICD9 410–414)	0.04 (-0.02 to 0.11)
Mayak workers CeVD	Azizova et al. [41–43]	0.51 (0 to > 4.5)^a^	External gamma	Smoking, BMI, hypertension, alcohol consumption	22,377 (425,735)	8717	Incidence	5	CeVD (ICD9 430–438)	0.46 (0.37 to 0.56)
								10	CeVD (ICD9 430–438)	0.49 (0.39 to 0.60)
								15	CeVD (ICD9 430–438)	0.53 (0.43 to 0.65)
		0.43 (< 0.1 to > 3)	Liver external gamma	Smoking, BMI, diabetes, alcohol consumption	22,377 (459,520)	9469		10	CeVD (ICD9 430–438)	0.39 (0.31 to 0.48)
		0.51 (0 to > 4.5)^a^	External gamma	Smoking, BMI, hypertension, alcohol consumption,	22,377 (836,078)	1578	Mortality	5	CeVD (ICD9 430–438)	0.05 (-0.04 to 0.16)
								10	CeVD (ICD9 430–438)	0.05 (-0.03 to 0.16)
								15	CeVD (ICD9 430–438)	0.06 (-0.03 to 0.18)
		0.43 (< 0.1 to > 3)	Liver external gamma	Smoking, BMI, diabetes, alcohol consumption	22,377 (890,132)	1808		10	CeVD (ICD9 430–438)	0.03 (-0.05 to 0.13)
Mayak workers all cardiovascular disease	Azizova et al. [41, 44]	0.51 (0 to < 4)	External gamma	Smoking, BMI, hypertension, alcohol consumption	22,334 (836,048)	5010	Mortality	5	CVD (ICD9 390–459) < 4 Gy	0.08 (0.02 to 0.14)
								10		0.08 (0.02 to 0.15)
								15		0.09 (0.03 to 0.16)
		0.43 (< 0.1 to > 3)	Liver external gamma	Smoking, BMI, diabetes, alcohol consumption	22,377 (890,132)	6019		10	CVD (ICD9 390–459)	0.03 (-0.01 to 0.09)
Mayak workers lower extremity arterial disease	Azizova et al. [45]	0.51 (0 to > 4.5)	External gamma	Smoking, BMI, hypertension, alcohol consumption	21,122 (512,801)	943	Incidence	5	Lower extremity arterial disease (ICD9 440.2)	0.30 (0.13 to 0.53)
								10	Lower extremity arterial disease (ICD9 440.2)	0.28 (0.12 to 0.50)
								15	Lower extremity arterial disease (ICD9 440.2)	0.32 (0.14 to 0.57)
**Environmental studies**										
Techa River study	Krestinina et al. [54]	0.034 (0 to 0.995)	Muscle	Ethnic group, settlement status	60,205 (1,836,203)	14,830	Mortality	5	CVD (ICD9 390–459)	0.12 (-0.70 to 0.32)
								10	CVD (ICD9 390–459)	0.19 (-0.50 to 0.40)
								15	CVD (ICD9 390–459)	0.30 (0.08 to 0.52)
						6163		5	IHD (ICD9 410–414)	0.64 (0.29 to 1.01)
								10	IHD (ICD9 410–414)	0.79 (0.42 to 1.19)
								15	IHD (ICD9 410–414)	0.92 (0.54 to 1.35)
						4388		5	CeVD (ICD9 430–438)	0.23 (-0.16 to 0.67)
								10	CeVD (ICD9 430–438)	0.30 (-0.09 to 0.76)
								15	CeVD (ICD9 430–438)	0.34 (-0.07 to 0.82)

*BMI* body mass index, *CeVD* cerebrovascular disease, *CI* Confidence Interval, *CVD* cardiovascular disease, *ICD* International Classification of Diseases, *IHD* ischemic heart disease

^a^doses given here are from Azizova et al. [40, 42]

## Results

Of the total of 93 studies from the original systematic review and meta-analysis (detailed in Little et al. [12]), 43 studies satisfied the a priori selection criteria and were retained for this analysis.

Of the 50 studies that were omitted, the high dose radiotherapeutic studies (of the type shown in Table 1) in many cases [55–79] had information on many lifestyle and environmental variables, but only presented one type of analysis (generally fully adjusted); however, there were some high dose studies in which there was little or no information on potential confounders [80, 81]. The Danish study of Lorenzen et al. [82] was omitted as it is largely subsumed by the Nordic study of Darby et al. [19]. There was a similar situation for the lower dose studies (of the type shown in Table 2), which in some cases had rich lifestyle information but only presented a single type of analysis [83–88] although in many instances the lower dose studies that were omitted had little or no information, apart from crude markers of socioeconomic status [54, 89–111]. Of the final selected studies, 22 were of groups exposed to substantial doses of medical radiation for therapy or diagnosis (Table 1). The remaining 21 studies were of groups exposed at much lower levels of dose and/or dose rate (Tables 2 and 3). There is substantial overlap in the populations studied in some of these groups. For example three of the studies relate to various CVD endpoints in the LSS [9, 10, 37], and there are various studies of a number of CVD endpoints in the Mayak nuclear workers [40–46].

### Effects of adjustment for potential confounding variables

Very few studies suggested substantial effects of adjustment for lifestyle, environmental, or medical risk factors. In the Rochester study of Adams et al. [34] the unadjusted ERR/Gy for coronary heart disease was 0.08 (95% confidence interval (CI) -0.01 to 0.20), and the ERR/Gy adjusted for sex, diabetes, dyslipidemia, smoking and hypertension was -0.03 (95% CI -0.07 to 0.10) (Tables 1 and 3). In the LSS the unadjusted ERR/Gy for hypertensive heart disease incidence was 0.01 (95% CI -0.08 to 0.10), and the ERR/Gy adjusted for smoking and drinking was -0.01 (95% CI -0.09 to 0.09) [9] (Tables 2 and 3). In the French uranium miners case–control study of Drubay et al. [48] the unadjusted ERR/Gy for all CVD was 0.4 (95% CI -1.8 to 3.0), and adjusted for smoking, body mass index (BMI), diabetes, hypertension, hypercholesterolemia, hypertriglyceridemia, resting heart rate, chronic kidney disease, hyperuricemia, gamma-glutamyl transpeptidase was -0.7 (95% CI -3.2 to 2.9) (Tables 2 and 3). The unadjusted ERR/Gy for hypertension, or CVD excluding cerebrovascular disease (CeVD) and some other endpoints in the Korean medical diagnostic workers were 0.3 (95% CI -6.9 to 9.6) and 1.3 (95% CI -5.0 to 9.0), whereas for the same endpoints adjusted for sex, smoking, alcohol intake, BMI, blood pressure, cholesterol and blood glucose the ERR/Gy were -1.8 (95% CI -8.3 to 6.8) and -0.5 (95% CI -6.3 to 6.7), respectively [51] (Tables 2 and 3).

### Modifying effects of radiation

There are only two studies reporting the most serious level of modifying effect, those of the International Nuclear Workers Study (INWORKS) workers by Gillies et al. [39] and the Korean medical diagnostic worker study of Cha et al. [51] (Tables 2 and 3). Gillies et al. [39] reported markedly higher risks for females, with ERR/Gy of 4.22 (90% CI 1.72 to 7.21) for all CVD, 6.17 (90% CI 2.44 to 10.92) for ischemic heart disease (IHD), and 2.67 (90% CI < 0 to 9.79) for CeVD, compared with ERR/Gy for males for the same endpoints of 0.20 (90% CI 0.07 to 0.36), 0.16 (90% CI -0.01 to 0.34), and 0.48 (90% CI 0.10 to 0.91), respectively; these differences were highly significant for all CVD (*p* = 0.005) and IHD (*p* = 0.004), but not for CeVD (*p* > 0.50) (Tables 2 and 3). Gillies et al. [39] also reported modifications by attained age and duration of employment, and although some of these were substantial for certain groups (Tables 2 and 3) none were statistically significant (*p* > 0.10). The Korean worker study of Cha et al. [51] also reported markedly higher ERR/Gy for females, with CVD ERR/Gy of 42.1 (95% CI 3.0 to 99.2) compared with ERR/Gy for males of -0.7 (95% CI -7.6 to 7.6). This study also reported significant modifications of ERR/Gy for CVD in relation to age, birth year, year started work and years worked (Tables 2 and 3). There were similar indications of heterogeneity for all these variables for hypertension and CVD excluding CeVD and other CVD (ICD10 I70-I83, I85-99). The French Childhood Cancer Study of Mansouri et al. [16] reported marked discrepancies with treatment status by anthracyclines (cardiotoxic anticancer drugs), with an ERR/Gy of 0.44 (95% CI 0.18 to 1.12) for those not treated compared with an ERR/Gy of 0.09 (95% CI 0.02 to 0.22) for those treated with anthracyclines (Table 1). They also reported that “HF [heart failure] risk increased with each category of age, with ERR/Gy values for the MHD [mean heart dose] of 0.06, 0.33, 0.38 and 0.48 in those aged < 15, 15–25, 25–35, and ≥ 35 years, respectively” [16]. The Netherlands-NKI-Rotterdam breast cancer case control study of Jacobse et al. [22] reported borderline significant modifications in risk by age at exposure (ERR/Gy age < 45 = 0.242 (95% CI 0.044 to 0.823), age 45–49 = 0.111 (95% CI 0.012 to 0.401), age 50–70 = 0.025 (95% CI -0.014 to 0.119), heterogeneity *p* = 0.07) (Table 1). In no other high radiation dose studies (reported in Table 1) were any modifying effects reported [17–19, 22, 23]. In the LSS, there are significant modifying effects on CeVD risk of attained age and age at exposure [10, 11], with ERR decreasing in each case with increases in each variable, but no such modifications in ERR by any of these variables when the investigators used only CVD as outcome; Little et al. [11] reported a change in ERR/Gy per year of age at exposure by -0.050 (95% CI -0.099, -0.015) for stroke and by -0.012 (95% CI -0.041, 0.018) for heart disease. There is a borderline significant effect (*p* = 0.022) of sex on heart disease but not for stroke (*p* > 0.9) [11].

Although not reported in Table 2, because the analysis was not generally aligned with that of the main analysis (using a lag of 5 years, and wherever possible dose < 4 Gy), there is analysis of the Mayak worker data by sex, attained age and duration of employment group; there was no statistically significant heterogeneity (*p* > 0.1) in effect suggested by these analyses [41, 42, 44–46].

### Modifying effects of latency

Table 4 shows the modifying effects of latency. There is very little variation of ERR/Gy with lag, although there is a slight tendency for ERR/Gy to increase with increasing lag period.

### Modifying effects in animal data

Table 5 illustrates what little is known about potential modifying factors from radiobiological animal data. There is some evidence of the modifying effects of age at exposure and chemotherapy in certain systems.

**Table 5:** Effect of modifying variables on absolute risk in radiobiological animal data

Reference	Animal model	Variable	Endpoints	Variable effect on radiation endpoints	Notes
**Sex**					
Sridharan et al. [112]	Adult mice, high activated protein C expressers, exposed to leg-out partial body γ-rays	Sex	Cardiac function, cardiac collagen deposition, cardiac microvascular density	Radiation endpoints differ between the sexes	No quantitative data on differences in relative radiation effect (irradiated vs not) by sex presented (but possibly could be derived from data provided in supplement). Only tissue level data presented
Stewart et al. [113]	Adult ApoE^–/–^ mice, exposed to aortic arch and carotid artery X-rays	Sex	Number of plaques, plaque histology	No sex difference in plaque burden, some differences in plaque immune cells	Few quantitative data on sex difference presented, and difficult to assess sex modifications of relative effects
Andruska et al. [114]	Adult Dahl SS rats, exposed to local heart X-rays	Sex	Cardiac function, pericardial effusions	Endpoints more severe in female rats, but may be due to a higher volume of irradiated lung. Female pericardial effusion prevalence significantly greater than in males among irradiated rats (*p* < 0.001), but male survival significantly worse (*p* < 0.01) than females. Various cardiac output measures suggest females have more rapid onset of cardiac dysfunction than males	Few quantitative data on sex difference presented, and difficult to assess sex modifications of relative radiation effects (irradiated vs control)
Chmielewski-Stivers et al. [115]	Adult wild-type mice, exposed to local heart X-rays	Sex	Survival, echocardiography, histopathology of the heart	Males exhibited lower survival than females. Males showed echocardiographic and histopathological changes, but not in females	Few quantitative data on sex differences presented, and difficult to assess sex modifications of relative radiation effects (irradiated vs control)
	Adult RhoB^–/–^ mice, exposed to local heart X-rays	Sex	Survival, echocardiography, histopathology of the heart	Females exhibited lower survival than males. Females showed histopathological changes, but not in males. Both sexes showed no echocardiographic changes	In males, RhoB^–/–^ mice were more radioresistant than wild-type counterparts. The reverse held true for females. As above, few quantitative data on sex differences presented, and difficult to assess sex modifications of relative radiation effects (irradiated vs control)
**Age at exposure**					
Lenarczyk et al. [116]	Male WAG/RijCmcr rats exposed to whole body X-rays at the age of 6 weeks or 6 months	Age at exposure	Blood levels of cardiovascular risk factors, perivascular fibrosis, systemic blood pressure	Different dose- and time-dependent effects on cardiovascular risk factors between young and old animals. Increasing age at exposure resulted in more strongly positive dose response for albumin (*p* = 0.0003), protein (*p* = 0.0014), AST (*p* = 0.0014), and alkaline phosphatase (*p* = 0.0003), but more negative dose response for cholesterol (*p* = 0.0008), HDL (*p* = 0.0030), triglycerides (*p* = 0.0333), BUN (*p* = 0.0068) and calcium (*p* = 0.0299). More severe radiation-induced perivascular fibrosis and blood pressure increase in young animals	Few quantitative data on age at exposure differences presented, and difficult to assess age at exposure modifications of relative radiation effects (irradiated vs control)
Mitchel et al. [117]	ApoE^–/–^, p53^+/–^ mice, exposed to whole body γ-rays at 8 weeks or 7 months of age	Age at exposure	Vascular lesion size, lesion frequency, serum total cholesterol	p53 heterozygosity alters the effects of radiation on all endpoints. The type of modification depends on age at exposure (8 weeks vs 7 months)	Higher age is associated with more severe atherosclerosis at the time of radiation. However, few quantitative data on age at exposure differences presented, and difficult to assess age at exposure modifications of relative radiation effects (irradiated vs control)
**Anti-cancer treatment**					
Fajardo et al. [118], Eltringham et al. [119]	Young adult New Zealand White rabbit, exposed to local heart X-rays and adriamycin	Anthracycline	Cardiac histopathology	Cardiac histopathology worse in combined treatment (radiation + adriamycin) group	Difficult to determine magnitude of differential effect on radiation response (irradiated vs control) of adriamycin
Myers and Lu [120] Yan et al. [121] Du et al. [122]	Adult mice, thorax exposure to X-rays, pretreated with anti-PD-1 antibody or control IgG	Anti-PD-1	Animal survival, cardiac function, cardiac cytokine levels, cardiac immune cell infiltration	All outcomes are worse in combined treatment group (radiation + anti-PD-1)	Sex of the mice unknown. Difficult to determine magnitude of differential effect on radiation response (irradiated vs control) of anti-PD-1 or control IgG
Seemann et al. [123]	Adult male mice, exposed to local heart irradiation and lapatinib	Lapatinib (tyrosine kinase inhibitor)	Cardiac function, Cardiac microvascular density, cardiac immune cell infiltration, cardiac fibrosis	Lapatinib reduced immune cell infiltration. No other modification of radiation effects	Difficult to determine magnitude of differential effect on radiation response (irradiated vs control) of lapatinib
Sridharan et al. [124]	Adult male Sprague–Dawley rats, exposed to local heart irradiation and sunitinib	Sunitinib (tyrosine kinase inhibitor)	Cardiac cell apoptosis, cardiac oxidative stress, mitochondrial swelling	Sunitinib reduced the effects of radiation on apoptosis, did not alter oxidative stress, and aggravated mitochondrial swelling	Difficult to determine magnitude of differential effect on radiation response (irradiated vs control) of sunitinib
Kotla et al. [125]	Adult LDLR^–/–^ mice on high fat diet and exposed to neck and thorax X-rays, followed by TAC and PARP inhibitor treatment	PARP inhibitor	Cardiac function, artery stenosis, artery wall thickness, perivascular fibrosis, Mac3^+^ cells (macrophages) in vascular lesions	PARP inhibition reduced the effects of radiation on cardiac function, artery stenosis, and Mac3^+^ cell number	High fat diet and TAC were used to induce atherosclerosis. Difficult to determine magnitude of differential effect on radiation response (irradiated vs control) of PARP inhibitor

*ApoE* apolipoprotein E, *AST* aspartate immunotransferase, *BUN* blood urea nitrogen, *HDL* high-density lipoprotein, *IgG* immunoglobulin G, *LDLR* low-density lipoprotein receptor, *PARP* poly(ADP-ribose) polymerase, *PD-1* programmed cell death protein 1, *TAC* thoracic aorta coarctation

## Discussion

We have assessed effects of confounding by lifestyle, environmental or medical risk factors on radiation-associated CVD, using data assembled for a recent systematic review [12]. We found only limited evidence that adjustment for potential confounding made substantial difference to risk estimates. Only in four studies, in a group treated in childhood for hemangioma [34], in the LSS [9] and in two groups of nuclear workers [48, 51], were the adjusted and unadjusted ERR substantially different (Tables 1, 2 and 3). However, it was hard to assess whether these variables were true confounders of the association between radiation and CVD; there were also substantial uncertainties in all of these studies, so that not much weight can be attached.

We also investigated evidence for modifying effects of these variables on CVD radiation dose response, again using data assembled for the systematic review [12]. There are fewer suggestions of the most serious level of modifying effect, with age at exposure modifications in the same direction reported in two studies [11, 16], although for different disease endpoints. In the study of Mansouri et al. [16] there were substantial modifying effects of anthracycline exposure (Tables 1, 2 and 3). In the LSS and in two groups of nuclear workers there are significant modifying effects of sex [11, 39, 51], although for discrepant endpoints. However, in most of the studies reported here no analysis has been reported of modifying effects of these or any other variable (Tables 1, 2 and 3). There is little variation of ERR with lagging period, although there is a slight tendency for ERR/Gy to increase with increasing lag period. (Table 4).

The radiobiological animal data has rather less information (Table 5). The metrics used are heterogeneous, and in general the internal dose trends (ERR/Gy) used in the epidemiological data given in Tables 1, 2 and 3 are not given. Indeed, in most studies there is only a single irradiated group, and the relative effects of the extra covariate on the radiation-associated relative risk (irradiated vs control) difficult to determine. Given the heterogeneity in endpoints used and in the animal systems employed one should probably not attach much weight to these findings.

Assessment of outcomes is a complication, as mortality outcomes could be less accurate than studies of incidence. In incidence studies, medical and lifestyle factors are more likely to be collected, as reported in our systematic review [12]. Pooling mortality and incidence data could explain part of the heterogeneity of the summarized results observed in the meta-analysis. Indeed, summarized risks were significantly higher for mortality endpoints compared with those of incidence in the meta-analysis [12]. An additional complication in many of the studies presented, particularly of the LSS [9–11, 37], groups of nuclear workers (IARC 15-country and INWORKS nuclear worker studies [38, 39], Mayak nuclear workers [40–42, 44–46]) is the overlap in subjects included, a feature also of the systematic review from which they were drawn [12]. It is very likely that there is inter-study heterogeneity of effect in the present study, reflecting the heterogeneity that was observed in the systematic review from which it is drawn [12]. Some part of the heterogeneity in the previous review is clearly driven by differences in endpoint sensitivity, by age at exposure, by dose and dose rate, and as noted above mortality vs incidence, but even after accounting for the effect of these heterogeneity remained [12]. Such heterogeneity complicates any causal interpretation of the results presented.

Confounding is likely to be specific to each study and the effects of adjustment could rarely be generalized to other studies. Residual confounding could be an issue, if the potential confounding variable is measured with substantial error. Effect modification is likely to be much more easily compared between studies, although the evidence assembled here does not suggest that even for such easily and reliably measured variables as sex and age at exposure there are consistent effects within studies (Tables 1, 2 and 3). These differences in modifying effect between cohort may reflect the play of chance, but it is also possible that there are underlying differences between the cohorts. Medical and lifestyle factors are differently available in the studies considered, with more detailed information among studies of radiotherapeutic exposure, where radiation doses are high to moderate. However, the number of patients included in these medical studies is generally rather small, limiting study of specific endpoints. In contrast, in studies on workers or in general population, generally few potential confounding variables can be collected as the large number of people recruited and the way of collecting the information does not usually allow such information to be obtained. In our systematic review [12], among the lower dose studies with detailed information on lifestyle factors and a large number of included people there were only a few occupational cohorts, principally the Mayak worker cohort [40–42, 44–46], the Semipalatinsk cohort [53], and, with a rather smaller number of people included, the French nuclear fuel cycle workers [47, 48] and the Korean radiation worker cohorts [51, 52]. For the purposes of maximizing statistical power, specific CVD outcomes are analyzed together, but potential confounders could act differently on the different outcomes and their specific effect could be unseen in a pooled analysis of heterogeneous outcomes.

As summarized in Tables 1, 2 and 3, in general many epidemiological studies now have quite rich lifestyle, environmental and medical information. As highlighted in the Results there are over 30 other studies that clearly have such cofactor information, although in all cases best use has not been made of this in the publications for the purposes of assessing effects of potential confounding factors or effect modifying factors.

A limitation of our analysis is that statistical significance cannot be attached to the difference made by potentially confounding factors, since the reported coefficients would necessarily be highly correlated, and from the published data this correlation is impossible to determine. We therefore judge that this has to remain as we describe it in the Methods, based simply on the size and sign of the coefficients. However, as we outline in the Methods, one of the criteria for risk modifying factors is based on statistical significance. Clearly the particular levels of magnitude we chose to determine the seriousness of potentially confounding variables, likewise the levels of difference made by risk modifying factors are both somewhat arbitrary. Another limitation of the analysis is the degree of overlap in two particular studies, specifically two of the most important and informative ones, the LSS [9, 10, 37] and the Mayak workers [40–46], although not in any of the other studies listed in Tables 1, 2, 3 and 4. However, given the form of the analysis, bias would not result from this. At most there would be a tendency for findings (of potential confounding or risk modification) to be inflated by these correlated findings. As may be inferred from the results presented in Tables 3 and 4, there is little evidence for this, although the Mayak worker data do show a similar direction of effect made by adjustment to two overlapping mortality endpoints, IHD and all CVD [41].

In many studies in which adjustment is made for certain covariates, these are assayed at a number of time points and this information is then used to adjust for health endpoints after that point. Some of these covariates may also affect competing risks, for example cancer, and it is possible that they may affect both baseline CVD risk as well as radiation-associated excess risk; however, the evidence we have presented (Tables 1, 2 and 3) does not suggest that this is likely. Competing risks may well not be independent of CVD, so that the censoring they introduce will be informative. In this case consideration may have to be given to non-standard ways of analyzing the data. There are a number of statistical methods to assess effects of two or more competing risks [126]. One of the most popular is the so-called subdistribution hazard of Fine and Gray [127].

In summary, because of the multifactorial etiology of CVD, medical and lifestyle factors are clearly crucial variables to take into account in analysis of the dose response of these endpoints. The heterogeneity of the studied populations and of the type of exposure and dosimetry complicates drawing conclusions on the impact of medical and lifestyle factors on the dose response relationship between exposure to radiation and CVD. Nevertheless, we found a large number of studies in which there is information on effect of adjusting for certain lifestyle/environmental/medical variables, although in the larger number of studies previously assessed this information was not available, even if the relevant variables had clearly been assessed. We found limited evidence of potential confounding of radiation effects on CVD (Tables 1, 2 and 3); substantial differences were made by adjustment in four studies, but the uncertainties in all cases were substantial, so that little weight can be attached. There is much less information on potential modifying variables of radiation effect; nevertheless there is some evidence of the effects of age at exposure and sex, although not always in the same endpoints in different studies. However, in most of the studies reported here no analysis was reported of modifying effects of these or any other variable (Tables 1, 2 and 3). There is little evidence of modification resulting from variation in lagging period (Table 4). Efforts should be made to include in future studies as much as possible precise information on these variables and if available specific analysis on their impact on the dose response relationship should be assessed. It is important that analyses of radiation-associated CVD clearly demonstrate the effect of adjustment for the available lifestyle/environmental/medical variables, and also assess the potential modifying effect of these variables on the radiation dose response.

## Data Availability

All data used are listed in Tables 1, 2, 3 and 4.
